# B and T lymphocyte attenuator (BTLA) and PD-1 pathway dual blockade promotes antitumor immune responses by reversing CD8^+^ T-cell exhaustion in non-small cell lung cancer

**DOI:** 10.3389/fimmu.2025.1553042

**Published:** 2025-05-20

**Authors:** Yang Zhang, Yang Yang, Yuanyuan Zeng, Qiuxia Qu, Dan Shen, Chuanyong Mu, Wei Lei, Meiqin Su, Jingyu Mao, Lirong Gao, Zeyi Liu, Cheng Chen, Jian-an Huang

**Affiliations:** ^1^ Department of Pulmonary and Critical Care Medicine, the First Affiliated Hospital of Soochow University, Suzhou, China; ^2^ Institute of Respiratory Diseases, Soochow University, Suzhou, China; ^3^ Clinical Immunology Laboratory, the First Affiliated Hospital of Soochow University, Suzhou, China

**Keywords:** NSCLC, BTLA, PD-1/PD-L1, immunotherapy, combination

## Abstract

**Background:**

Immunotherapies targeting the programmed cell death 1 (PD-1)/programmed death ligand 1 (PD-L1) have shown great promise for a subset of patients with non-small cell lung cancer (NSCLC). However, safe and robust combination therapies are still needed to bring the benefit to broader patient populations.

**Methods:**

we performed *in vivo* treatment with PD-L1 antibody in Lewis lung carcinoma (LLC)-derived murine NSCLC model. Expression of B and T lymphocyte attenuator (BTLA) was detected during treatment. We evaluated the effects of the combination of anti-BTLA and anti-PD-L1 mAbs on tumor growth and overall survival of mice. In addition, distribution and function of immune cells were analyzed by flow cytometry. The role of BTLA in human and murine CD8^+^ T cells and its impact on reversing exhausted phenotype of PD-1^+^CD8^+^ T cell by PD-L1 blockade were analyzed. Furthermore, we investigated expression and distribution of BTLA on lymphocytes in tumor microenvironment of different specimens from NSCLC patients.

**Results:**

There was no significant difference overall survival between anti-PD-L1 therapy and IgG in LLC-bearing mice, and BTLA expression was increased on CD8^+^ T cells after PD-L1 antibody treatment. LLC-bearing mice treated with combination of anti-BTLA and anti-PD-L1 therapy had an improved overall survival than anti-BTLA or anti-PD-L1 alone. Compared to monotherapy with anti-BTLA or anti-PD-L1, mice treated with combination therapy demonstrated increased infiltration of CD8^+^ and CD4^+^ T cells, as well as increased expression of IFN-γ, TNF-α and Ki-67 in CD8^+^ T cells. In addition, CD8^+^ T cells co-expressing BTLA and PD-1 exhibited the most exhausted phenotype to resist PD-L1 blockade therapy. Furthermore, BTLA^+^CD8^+^ T cells were abnormally increased in different specimens from NSCLC patients, and CD8^+^ T cells expressing BTLA in NSCLC microenvironment were correlated with clinical response to anti-PD-1 therapy in NSCLC patients.

**Conclusion:**

Our results show that BTLA and PD-1 cooperatively inhibit the activity of CD8^+^ T cells and are associated with resistance to PD-1/PD-L1 pathway blockade in NSCLC patients. Anti-BTLA blockade enhances the antitumor efficacy of anti-PD-L1 therapy. Dual BTLA and PD-1/PD-L1 blockade should be further explored to elicit potent antitumor CD8^+^ T-cell responses in NSCLC patients.

## 1 Introduction

Lung cancer is the leading cause of cancer incidence and mortality worldwide (1). Approximately 85% of these cases are non-small cell lung cancer (NSCLC), and most are diagnosed at an advanced stage (2). Immunotherapy can reverse tumor immune escape by suppressing immune checkpoints, and immune checkpoint blockade with antibodies against programmed death 1 (PD-1) and programmed death ligand 1 (PD-L1) has shown promise as an immunotherapy treatment strategy for NSCLC patients (3–5). Multiple immune checkpoint inhibitors targeting PD-1/PD-L1 have been approved by the FDA for first- and second-line treatment of NSCLC patients (6, 7). However, limited efficacy has been reported in PD-1/PD-L1 blockade therapy, and a large number of patients show partial responsiveness (4, 8), highlighting the necessity of further investigation into alternative strategies for those refractory to PD-1/PD-L1 blockade therapy.

In addition to PD-1, many other immune cell surface coinhibitory molecules regulate tumor immunity. tumor-infiltrating lymphocytes (TILs) co-expressing PD-1 and CTLA-4 or PD-1 and TIM-3 are functionally exhausted compared to other TIL subsets, and double blockade of these molecules and PD-1/PD-L1 increases the recovery of effector function (9–11). Several studies have shown that the combination of an anti-PD-1 antibody and an anti-CTLA-4 or anti-TIM-3 antibody provides more potent antitumor efficacy and significantly longer survival than any of these single antibodies alone in cancer patients (12–14). Therefore, such combinations involving immune checkpoint molecule antibodies provide promising strategies for NSCLC patients who have failed treatment with PD-1/PD-L1 inhibitors.

B and T lymphocyte attenuator (BTLA) belongs to the CD28 superfamily and is structurally and functionally similar to PD-1 as a coinhibitory checkpoint molecule (15). BTLA is widely expressed on the surface of various immune cells, such as T cells, B cells, monocytes, and natural killer (NK) cells (16, 17). BTLA is highly expressed on tumor-specific T cells in cancer patients, and its upregulation in several TILs is associated with poor prognosis (18–20). Herpes virus entry mediator (HVEM), the ligand for BTLA, was found to be overexpressed in NSCLC patients with lymph node metastasis (21). Previous studies have shown that BTLA and PD-1 are co-expressed in human melanoma CD8^+^ T cells and hepatocellular carcinoma CD4^+^ T cells (22, 23), and both BTLA and PD-1 can recruit SH2-containing protein tyrosine phosphatase (SHP)-1 and SHP-2 to suppress T-cell receptor signaling (24, 25). However, whether BTLA expression in the NSCLC microenvironment contributes to resistance to PD-1/PD-L1 blockade therapy and whether combined blockade of BTLA could improve the efficacy of PD-1/PD-L1 inhibitors are unclear.

In this study, we examined BTLA expression during treatment in a mouse model of LLC tumor burden that is insensitive to PD-1/PD-L1 blockade treatment. In addition, we analyzed the effects of combined blockade of BTLA and PD-1 on tumor growth and microenvironment. The effects of different BTLA and PD-1 expression patterns on CD8^+^ T cells function were investigated. Furthermore, we examined the expression characteristics of BTLA in NSCLC microenvironment and analyzed the correlation between BTLA expression and clinical response to PD-1 blockade therapy.

## 2 Methods

### 2.1 Patients

Patients with lung cancer were admitted to the First Affiliated Hospital of Soochow University from 2019 to 2022, with approval from the Ethics Committee of the First Affiliated Hospital of Soochow University (approval no. 2018-255) and written informed consent from each patient. The patients were histopathologically proven NSCLC and had not been treated with anti-PD-1/PD-L1 antibodies. The medical records were reviewed, and data regarding clinicopathologic features and treatment outcome were extracted (**Supplementary Table S1**). The objective response to treatment with anti-PD-1 antibodies, that is, including nivolumab and pembrolizumab, was evaluated according to the Response Evaluation Criteria in Solid Tumors, ver. 1.1. The tumor response was evaluated by a physician every 2 to 3 months by using CT. Progression-free survival (PFS) was defined as the interval from the initiation of anti-PD-1 antibody therapy to tumor progression or death without evidence of progression. Patients without documented clinical or radiographic disease progression or who were still alive were censored on the date of last follow-up.

### 2.2 Clinical samples

Tumor tissues, malignant pleural effusions and peripheral blood from NSCLC patients were derived from the First Affiliated Hospital of Soochow University. All human biological samples were collected after ethical approval of the studies by the local ethics committee and after written informed consent of the patients was obtained and in line with Good Clinical Practice guidelines.

Lung cancer tumor samples were mechanically dissociated and digested using accutase (PAA Laboratories or Sigma-Aldrich), collagenase IV (Worthington), hyaluronidase (Sigma-Aldrich), and deoxyribonuclease (DNase) type IV (Sigma-Aldrich) within several hours after excision. After 20 to 30 min of digestion at 37°C on a rotator, tumor-derived cells were carefully mashed through a 70-μm cell strainer and washed two times with cold phosphate-buffered saline (PBS; centrifugation, 250g, 10 min, 4°C). Red blood cell lysis was performed before cells were analyzed by fluorescence-activated cell sorting (FACS). Peripheral blood mononuclear cells (PBMCs) and pleural effusion mononuclear cells (PEMCs) were isolated from patient-derived blood and malignant pleural effusion using density gradient centrifugation (450g, 30 min at room temperature without brake) over Histopaque-1077 (Sigma-Aldrich). Human PBMCs and PEMCs were isolated from the interphase and washed several times with Dulbecco’s PBS (DPBS). After red blood cell lysis, PBMCs and PEMCs were analyzed using FACS.

### 2.3 Cell lines

A549, H1299, H226 and HCC827 cells were obtained from the Cell Bank of the Chinese Academy of Sciences (Shanghai, China). Mouse lung cancer cell line LLC cells were obtained from the American Type Culture Collection (ATCC, Manassas, VA, USA). The cells were cultured in RPMI 1640 medium with 10% foetal bovine serum (Gibco, Carlsbad, CA, USA) and L-glutamine (Invitrogen, Carlsbad, CA, USA) at 37°C in a 5% CO_2_.

### 2.4 Mice

Female C57BL/6J mice (aged 6–8 weeks) were purchased from the Vital River Laboratory Animal Technology Co., Ltd (Beijing, China). All mice were bred in the animal facility of the Soochow University. All animal procedures were performed in accordance with approved protocols, and the approval number for this study is 2018-255. Animals were housed under specific pathogen-free conditions, maintained on a 12-hour light/dark cycle, and provided ad libitum access to food and water throughout the study.

### 2.5 *In vivo* tumor treatment

Anti-BTLA mAb (clone PJ196, Bio X cell), anti-PD-L1 mAb (clone 10F.9G2, Bio X cell) and IgG (clone MOPC-21, Bio X cell) were used for the following experiments. Briefly, C57BL/6J mice were subcutaneously challenged with 2×10^5^ LLC cells on day 0. Tumor bearing mice were randomized into four treatment cohorts: control IgG, anti-BTLA mAb, anti-PD-L1 mAb or anti-BTLA mAb combined with anti-PD-L1 mAb. All antibodies were administered at a dose 150 μg/mouse through intraperitoneal injection every 2 days for a total of three times on 4-5d after tumor inoculation. 24 hours after the last antibody treatment, the tumors were harvested and processed for flow cytometry analysis, and the remaining animals were kept until 50 days after tumor challenge or tumor volume reached 2 cm^3^ for survival analysis. Tumor growth was measured every second or third day, and tumor volume was calculated as (length×width×width)/2.

### 2.6 Co-culture and antibody blockade

CD8^+^T cell were isolated from LLC tumor-bearing C57BL/6 mice using MojoSort Mouse CD8 T Cell Isolation Kit (BioLegend). Purity (>90%) was confirmed by flow cytometry. 1x10^6^ CD8^+^ T cells were co-cultured with 1x10^5^ LLC cells (labeled CFSE) in 6-well plate with medium including anti-CD3/CD28 beads (1 ug/ml). 48 hours of 20ug/ml anti-BTLA, PD-L1 mAbs or combined treatment, co-cultured cells were stained with Annexin V/PI to analyze target cell apoptosis by gating on labeled LLC cells.

### 2.7 CD8^+^ T cell depletion study

LLC tumor was confirmed in mice on post implantation day 4. After randomization, tumor bearing mice received IP injections of anti-CD8 (clone 53-6.7, Bio X Cell, catalog#: BE0004-1) at 150 μg/dose. Control mice received 150 μg IgG IP (clone 2A3, Bio X Cell, catalog#: BE0089). CD8 depletion antibody were administered 24 hours prior to administration of the first dose of anti-BTLA and PD-L1 combined treatment (120 μg/dose). Depletion antibodies were administered every seven days and tumor volumes were measured every two days until day 28 post-implantation. T cell depletion was confirmed via flow cytometry of spleen in each treatment arm.

### 2.8 Tissue processing and flow cytometry

24 hours after the last antibody treatment, mice were sacrificed and tumors were isolated. Isolated tumors were cut into small pieces and digested with 0.25mg/ml Liberase TL (Roche) and 0.33mg/ml Dnase (Sigma) in 37°C for 30 min. Obtained single-cell suspensions were stained with BTLA, PD-1, CD45, CD3, CD4, CD8, F4/80 or CD11b mAbs at 4°C for 30 min. To stain for nuclear expression Foxp3 and Ki67, cells were fixation permeabilization with Fixation and Permeabilization buffer (eBioscence) for 30 min. For IFN-γ or TNF-α staining, cells were stimulated for 6 h with 50ng/ml phorbol 12-myristate 13-acetate (PMA) and 1μg/ml ionomycin (Sigma-Aldrich) in the presence of 10μg/ml Brefeldin A. After stimulation, cells were stained for antibodies to surface marker, followed by fixation permeabilization with Fixation and Permeabilization buffer according to the manufacturer’s instructions. Then, cells were stained with antibodies to intracellular markers. All the antibodies were purchased from BioLegend. Flow cytometric analyses were performed using a BD FACSCanto II flow cytometer (BD Bioscience) with FlowJo software (Tree star).

### 2.9 Immunohistochemistry

For IHC staining, the sections underwent antigen retrieval by heating in sodium citrate buffer (Bioss). Endogenous peroxidase activity was blocked with 3% hydrogen peroxide in methanol. Subsequently, the sections were washed with phosphate-buffered saline (PBS) three times and blocked with 5% bovine serum albumin (BSA) at 37°C for 1hour to reduce non-specific binding. Next, the sections were incubated with primary antibodies at 4°C overnight. The primary antibodies used in this study included HVEM (1:200, Abcam). The appropriate secondary antibodies were applied, followed by incubation with streptavidin–biotin complex (SABC) using the SABC immunohistochemical kit (BOSTER). Subsequently, the sections were subjected to DAB staining (BOSTER) to visualize the specific antigen–antibody complexes. To quantify the IHC staining results on patients with NSCLC, the IHC staining intensity was assigned scores: 0 (negative), 1 (weak), 2 (medium), or 3 (strong), and IHC score was calculated by multiplying the percentage of positive cells (P) by the intensity (I), using the formula: Q=P×I.

### 2.10 Statistical analysis

Data (mean ± SEM) are representative of independent experiments. Group mean comparisons were performed using GraphPad Prism software, V.8.4.3. We used the two-tailed unpaired Student’s t-test, Mann–Whitney U-test, or the log-rank test (survival studies). *P* < 0.05 was considered as statistically significant. Bars represent mean ± SEM. Asterisk coding is indicated as ^∗^ *P* < 0.05; ^∗∗^ *P* < 0.01; ^∗*∗^ *P* < 0.001; n.s. denotes not significant.

## 3 Results

### 3.1 Anti-PD-L1 blockade up-regulates expression of BTLA in CD8^+^ T cells in LLC-bearing mice

**Figure 1:**
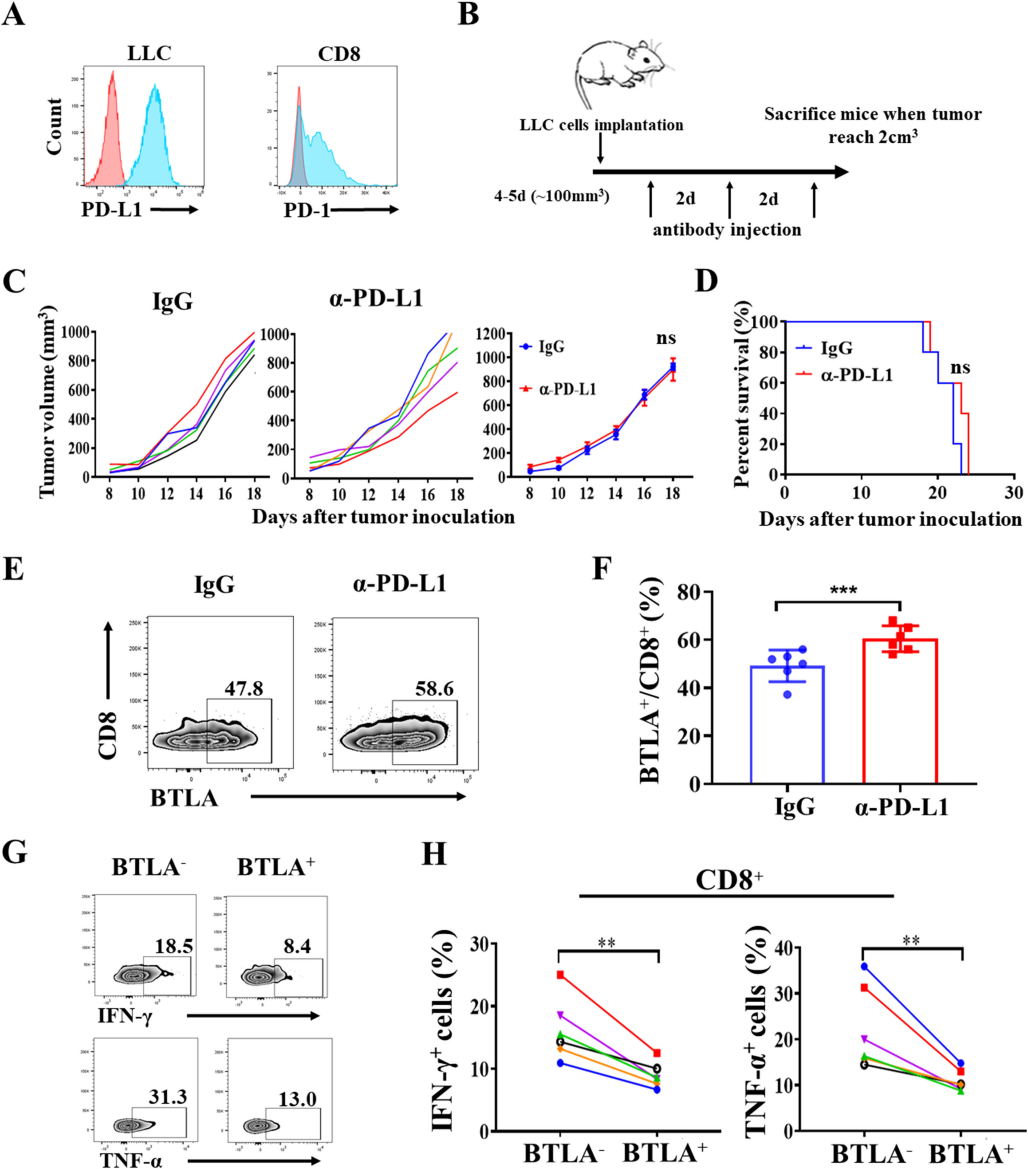
Anti-PD-L1 blockade up-regulates expression of BTLA in CD8^+^ T cells in LLC-bearing mice. **(A)** Representative flow cytometric analysis of PD-L1 expression on LLC cells, and PD-1 expression on CD8 cells in C57BL/6J mice LLC transplanted tumor. **(B)** Schematic representation of experimental setup of anti-PD-L1 antibody treatment. **(C)** Tumor growth curves of LLC tumor-bearing mice after treatment with control IgG and anti-PD-L1 mAb and mean tumor volumes of LLC tumor-bearing mice treated with indicated antibody (5 mice in each group). *P* values were calculated by one-way ANOVA test. **(D)** Overall survival of LLC tumor-bearing mice treated with indicated antibody (5 mice in each group). Differences in survival curves between groups were analyzed using the log-rank (Mantel – Cox) test. **(E, F)** Statistical analysis of BTLA expression in CD8^+^ T cells from tumor-infiltrating lymphocytes of LLC-bearing mice within 24h of the last antibody injection. n=6, paired student’s t-test was performed as statistical analysis. **(G, H)** Statistical analysis of IFN-γ and TNF-α expression in BTLA^-^ and BTLA^+^CD8^+^ T cells from tumor-infiltrating lymphocytes of LLC-bearing mice. n=6, Comparison between two groups is made via the unpaired student’s t-test. Survival was analyzed via Kaplan-Meier method and compared by log-rank (Mantel-Cox) text. (**, *P* < 0.01; ***, *P* < 0.001; ns, *P* > 0.05).

To investigate whether BTLA is involved in resistance to PD-1/PD-L1 blockade therapy, we performed *in vivo* treatment with PD-1/PD-L1 antibodies in Lewis lung carcinoma (LLC)-derived murine NSCLC model. Previous studies reported that LLC-derived murine NSCLC model was resistant to PD-1/PD-L1 blockade therapy (26). We first confirmed that LLC cells expressed PD-L1 and tumor-infiltrating CD8^+^ T cells expressed PD-1 in the transplanted tumors (**Figure 1A**). Tumor-bearing mice were intraperitoneally injected with 15 mg/kg anti-PD-L1 mAb or the isotype IgG control three times per week (**Figure 1B**). We found that the administration of anti-PD-L1 mAb had no effect on tumor growth than IgG control (*P* > 0.05) (**Figure 1C**). Compared to the control, overall survival was not improved with the anti-PD-L1 mAb treatment (*P* > 0.05) (**Figure 1D**). Then, the BTLA expression within 24h of the last antibody injection was analyzed. We found that BTLA expression was increased on CD8^+^ T cells after PD-L1 antibody treatment (**Figures 1E, F**). In addition, IFN-γ and TNF-α were reduced in BTLA^+^CD8^+^ T cells compared to BTLA^-^CD8^+^ T cells (**Figures 1G, H**). Collectively these data suggest that up-regulation of BTLA expression during the treatment process led to the reduction of CD8^+^ T cell cytotoxic function, which may induce resistance to PD-L1 blockade in LLC mouse model.

### 3.2 BTLA antibody addition overcomes resistance to PD-L1 blockade

**Figure 2:**
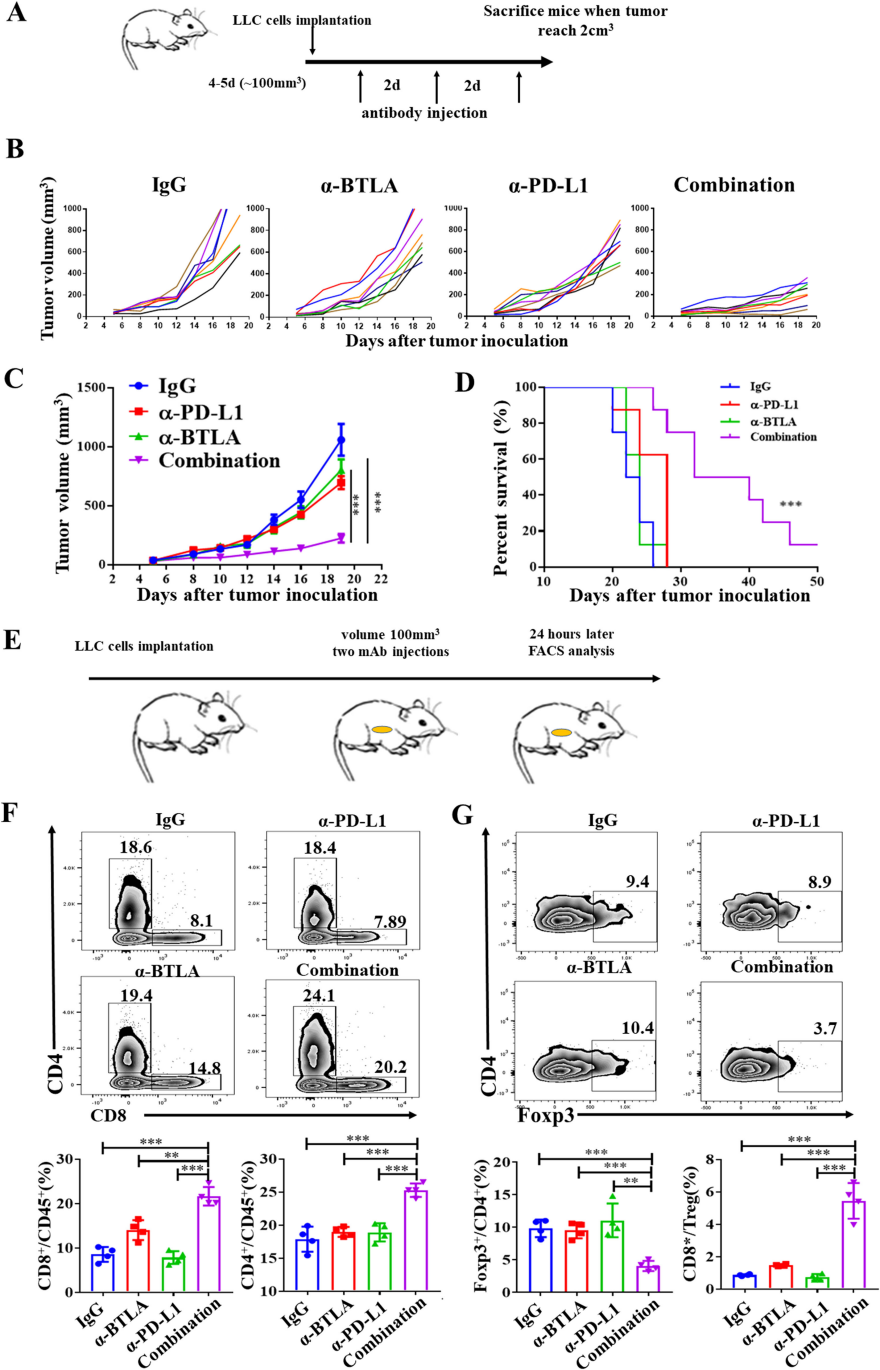
BTLA antibody addition overcomes resistance to PD-L1 blockade. **(A)** Schematic representation of *in vivo* experiment in mice treated with anti-BTLA, anti-PD-L1 single antibody or combination. **(B)** Tumor growth curves of LLC tumor-bearing mice after treatment with control IgG, anti-BTLA mAb, anti-PD-L1 mAb, or anti-BTLA mAb combined with anti-PD-L1 mAb (8 mice in each group). **(C)** Mean tumor volumes of LLC tumor-bearing mice treated with indicated antibodies (8 mice in each group). *P* values were calculated by one-way ANOVA. **(D)** Overall survival of LLC tumor-bearing mice treated with indicated antibodies (8 mice in each group). Differences in survival curves between groups were analyzed using the log-rank (Mantel – Cox) test. **(E-G)** LLC-bearing mice were treated with control IgG, anti-BTLA mAb, anti-PD-L1 mAb, or anti-BTLA mAb combined with anti-PD-L1 mAb starting on day 5 after tumor inoculation (150 μg/mouse), 24 h later, tumor-infiltrating lymphocytes were analyzed by flow cytometry. **(F)** Representative flow plots and statistics of percentages of CD8^+^ and CD4^+^ T cells (4 mice in each group). **(G)** Representative flow plots and statistics of percentages of Foxp3^+^CD4^+^ T and the radio of CD8^+^ T cells to Treg; (4 mice in each group). Comparison between two groups is made via the unpaired student’s t-test. Survival was analyzed via Kaplan-Meier method and compared by log-rank (Mantel-Cox) text. (**, *P* < 0.01; ***, *P* < 0.001).

To further validate the role of BTLA in PD-L1 blockade, we observed whether anti-BTLA treatment reversed resistance to PD-L1 blockade therapy in LLC mouse model. Tumor-bearing mice were intraperitoneally injected with 15 mg/kg anti-BTLA mAbs, anti-PD-L1 mAbs or the isotype IgG control three times per week (**Figure 2A**). We found that no statistically significant difference in tumor growth between anti-BTLA or anti-PD-L1 mAbs alone and control group, whereas combined treatment with anti-BTLA and anti-PD-L1 mAbs resulted in slower tumor growth than treatment with anti-BTLA or anti-PD-L1 mAbs alone (**Figures 2B, C**). Compared to the control, neither the anti-BTLA nor the anti-PD-L1 mAbs mediated long-term survival in LLC tumor model. However, combination treatment with dual checkpoint blockade resulted in improved overall survival (**Figure 2D**). In addition, we evaluated the effects of the combination of anti-BTLA and anti-PD-L1 mAbs on infiltrating T lymphocytes in LLC tumor model. We found that 24 h after two rounds of antibody administration (**Figure 2E**), the combination of anti-BTLA and anti-PD-L1 mAbs significantly increased the tumor infiltration of CD8^+^ and CD4^+^ T cells compared to that in the other groups (**Figure 2F**). Furthermore, in the combination group, the proportion of Treg cells was significantly reduced, and the ratio of CD8^+^ T cells to Treg cells was increased (**Figure 2G**). These results suggest that anti-BTLA blockade enhances the antitumor efficacy of anti-PD-L1 blockade in an anti-PD-L1 therapy-resistant tumor model, and dual BTLA and PD-1 blockade could increase CD8^+^ T cell infiltration and decreases Treg cell infiltration.

### 3.3 Combination of anti-BTLA blockade with PD-L1 inhibition induces CD8^+^ T-cell antitumor efficacy

**Figure 3:**
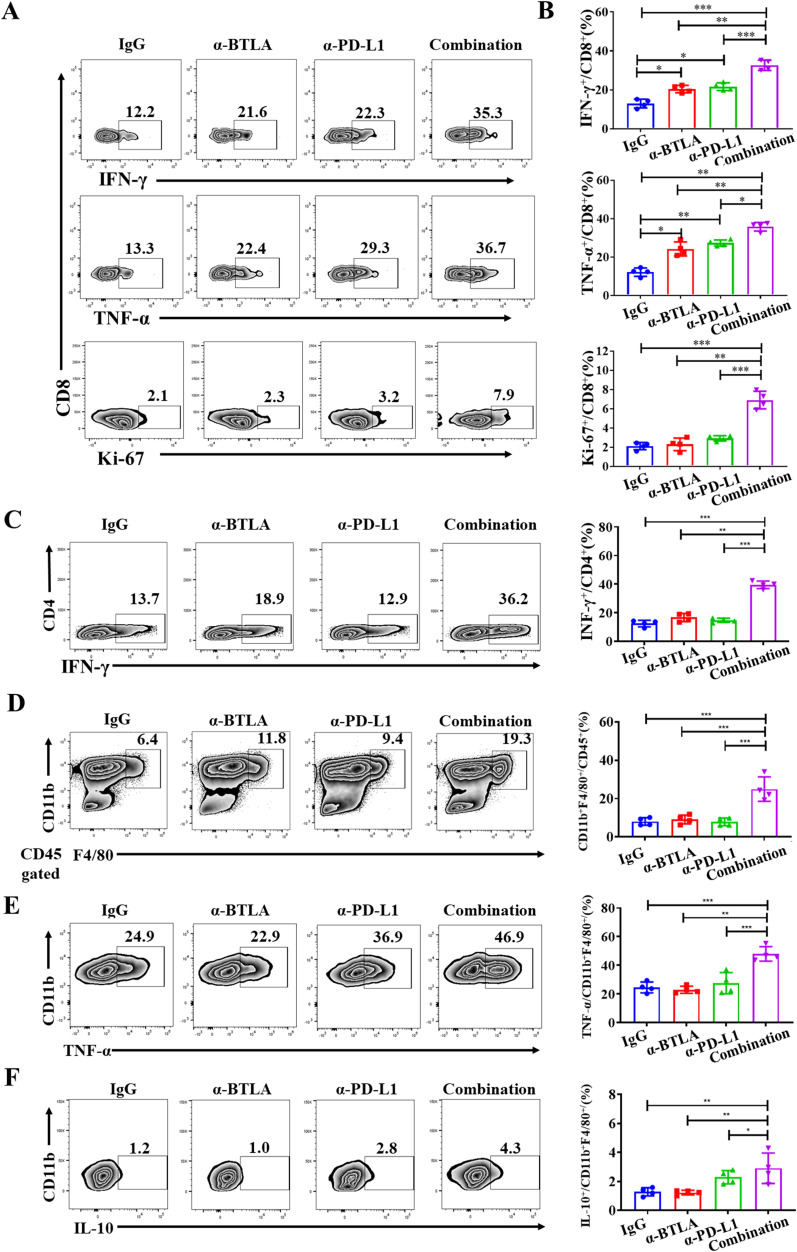
Combination of anti-BTLA blockade with PD-L1 inhibition induces CD8^+^ T-cell antitumor efficacy. LLC-bearing mice were treated with control IgG, anti-BTLA mAb, anti-PD-L1 mAb, or anti-BTLA mAb combined with anti-PD-L1 mAb, 24 h later, tumor-infiltrating lymphocytes were analyzed by flow cytometry. **(A, B)** CD8^+^ T cells were assessed by flow cytometry for expression of IFN-γ, TNF-α and Ki-67. **(C)** CD4^+^ T cells were assessed by flow cytometry for expression of IFN-γ. **(D)** Proportion of CD11b^+^F4/80^+^ macrophages in tumor were assessed by flow cytometry. **(E, F)** CD11b^+^F4/80^+^ macrophages were assessed by flow cytometry for expression of TNF-α and IL-10. Unpaired student’s t-test was performed as statistical analysis. (*, *P* < 0.05; **, *P* < 0.01; ***, *P* < 0.001).

We further analyzed the effect of combined anti-BTLA and anti-PD-L1 mAbs on lymphocyte function in tumor immune microenvironment. We found that 24 h after two rounds of antibody administration, the production of IFN-γ and TNF-α by CD8^+^ T cells in the anti-BTLA and anti-PD-L1 groups was greater than IgG control group. Moreover, the CD8^+^ T cells in the combination group produced more IFN-γ and TNF-α than other groups. In addition, the proportion of Ki67^+^CD8^+^ T cells was also significantly greater in the combination group than other groups (**Figures 3A, B**). Similarly, we also found the production of IFN-γ by CD4^+^ T cells in the combination group was higher than other groups. There was no significant difference between anti-BTLA or anti-PD-L1 group and control group (**Figure 3C**). Likewise, the proportion of CD11b^+^F4/80^+^ macrophage was also significantly greater in the combination group than other groups. There was no significant difference between anti-BTLA or anti-PD-L1 group and control group (**Figure 3D**). However, the proportion of M1 macrophages (TNF-α^+^CD11b^+^F4/80^+^) and M2 macrophages (IL-10^+^CD11b^+^F4/80^+^) increased simultaneously in the combination group (**Figures 3E, F**). These data demonstrated that combined immunotherapy with BTLA and PD-L1 mAbs might exert anti-tumor synergies by enhancing CD8^+^ T cell effector function in tumor microenvironment.

### 3.4 CD8^+^ T cells coexpressing BTLA and PD-1 exhibited the most exhausted phenotype to resist PD-L1 blockade therapy

**Figure 4:**
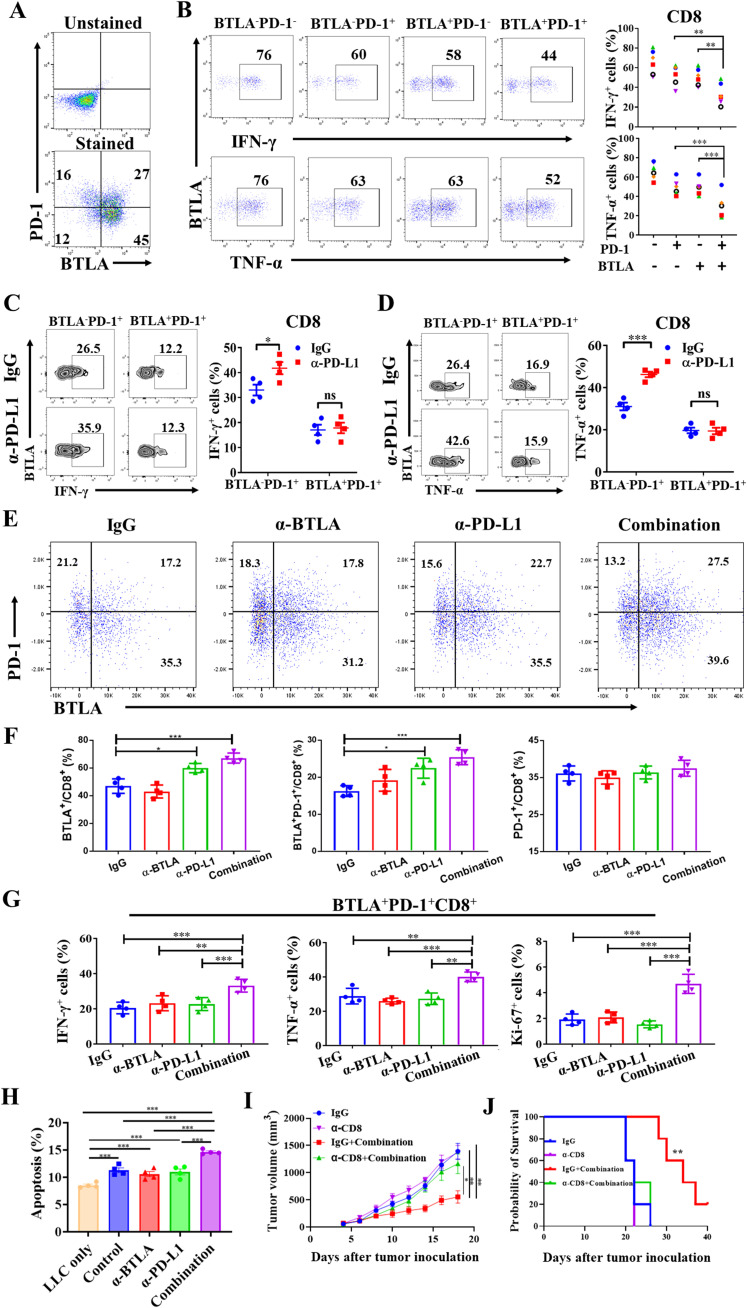
CD8^+^ T cells coexpressing BTLA and PD-1 exhibited the most exhausted phenotype to resist PD-L1 blockade therapy **(A, B)** Tumors were harvested from LLC-bearing mice on day 8 after tumor inoculation. **(A)** CD8^+^ T cells were assessed by flow cytometry for expression of PD-1 and BTLA. **(B)** BTLA^-^PD-1^-^, BTLA^-^PD-1^+^, BTLA^+^PD-1^-^ and BTLA^+^PD-1^+^CD8^+^ T cells were assessed by flow cytometry for expression of IFN-γ and TNF-α, 6 mice in each group. *P* values were calculated by two-tailed paired Student’s t-test. **(C, D)** LLC-bearing mice were treated with control IgG and anti-PD-L1 mAb starting on day 5 after tumor inoculation (150 μg/mouse), 24 h later, tumor-infiltrating lymphocytes were analyzed by flow cytometry, 4 mice in each group. BTLA^-^PD-1^+^ and BTLA^+^PD-1^+^CD8^+^ T cells were assessed by flow cytometry for expression of IFN-γ and TNF-α. **(E-G)** LLC-bearing mice were treated with control IgG, anti-BTLA mAb, anti-PD-L1 mAb, or anti-BTLA mAb combined with anti-PD-L1 mAb starting on day 5 after tumor inoculation (150 μg/mouse), 24 h later, tumor-infiltrating lymphocytes were analyzed by flow cytometry, 4 mice in each group. **(E, F)** CD8^+^ T cells in each antibody treatment group were assessed by flow cytometry for expression of PD-1 and BTLA. **(G)** BTLA^+^PD-1^+^CD8^+^ T cells in each antibody treatment group were assessed for expression of IFN-γ, TNF-α and Ki67. **(H)** Flow cytometric analysis of LLC apoptosis following 48h co-culture with CD8^+^ T cells under BTLA and PD-L1 antibody blockade. **(I, J)** Tumor growth and survival analysis of tumor-bearing mice treated with anti-PD-1 and anti-PD-L1 combination therapy in the presence or absence of CD8^+^ T cell depletion. 5 mice in each group. Comparison between two groups is made via the unpaired student’s t-test. Survival was analyzed via Kaplan-Meier method and compared by log-rank (Mantel-Cox) text. (*, *P* < 0.05; **, *P* < 0.01; ***, *P* < 0.001; ns, *P* > 0.05).

Although the combination of anti-BTLA and anti-PD-L1 mAbs increased the antitumor effect of CD8^+^ T cells, the mechanism by which BTLA moderates the antitumor effects of PD-L1 blockade is unclear. Since the reversal of exhausted immunophenotype in PD-1^+^CD8^+^ T cell is main mechanism of PD-1/PD-L1 blockade in anti-tumor immunotherapy, we observed the effect of BTLA on reversal of PD-1^+^CD8^+^ T cell exhaustion under PD-1/PD-L1 blockade. There were approximately 20% of CD8^+^ TILs co-expressed BTLA and PD-1 in LLC-bearing mice (**Figure 4A**). In addition, BTLA^+^PD-1^+^CD8^+^ T cells exhibited greater impairment of IFN-γ and TNF-α production than BTLA^+^PD-1^-^CD8^+^ T cells and BTLA^-^PD-1^+^CD8^+^ T cells (**Figure 4B**). As shown in **Figures 4C, D**, compared with IgG control, BTLA^-^PD-1^+^CD8^+^ T cells secreted more IFN-γ and TNF-α under PD-L1 antibody treatment. However, there was no statistically significant difference in IFN-γ and TNF-α secretion in BTLA^+^PD-1^+^CD8^+^ T cells under treatment with PD-L1 antibody compared to IgG. Because BTLA and PD-1 act synergistically to maintain CD8^+^ T-cell dysfunction, blocking PD-L1 alone does not induce an immune response by reversing the PD-1^+^CD8^+^ T-cell exhaustion phenotype. Furthermore, we observed anti-BTLA treatment did not affect the expression of either PD-1 or BTLA on CD8^+^ T cells. However, an increased expression of BTLA and proportion of BTLA^+^PD-1^+^CD8^+^ T cells after anti-PD-L1 mAb treatment compared to IgG treatment (**Figures 4E, F**). We next evaluated the effect of combining BTLA and PD-L1 mAbs on BTLA^+^PD-1^+^CD8^+^ T cells. We found that after combination treatment of BTLA and PD-L1 mAbs, the secretion of IFN-γ and TNF-α in BTLA^+^PD-1^+^CD8^+^ T cells was significantly higher than BTLA or PD-L1 mAbs treatment alone. Similarly, the expression of Ki67 in BTLA^+^PD-1^+^CD8^+^ T cells was significantly increased in combination group than other groups (**Figure 4G**). The co-culture experiments further confirmed that the anti-tumor effect of combined blockade was mediated specifically by CD8^+^ T cells, As shown in **Figure 4H**, compared with the LLC only group, co-culture with CD8^+^ T cells significantly enhanced LLC cell apoptosis. While treatment with anti-BTLA or anti-PD-L1 mAb alone showed no statistically significant differences in apoptosis rates compared to the control group, the combination blockade group demonstrated a markedly increased proportion of apoptotic LLC cells relative to single antibody treatment groups. We conducted *in vivo* CD8^+^ T cell depletion experiments to further verify that the anti-tumor effect of combination therapy depends on CD8^+^ T cells. The results showed no tumor growth and survival difference between the CD8^+^ T cells depletion group, CD8^+^ T cells depleted plus combination therapy group, and IgG control group. However, the combination therapy without CD8^+^ T cells depletion exhibited significantly slower tumor growth and longer survival compared to both the IgG control group and the combination therapy plus CD8^+^ T cells depletion group (**Figures 4I, J**). Collectively these data suggest that CD8^+^ T cells co-expressing BTLA and PD-1 exhibited the most exhausted phenotype to resist PD-L1 blockade therapy.

### 3.5 Abnormally increased infiltration of BTLA^+^CD8^+^ T cells in NSCLC tumor microenvironment

**Figure 5:**
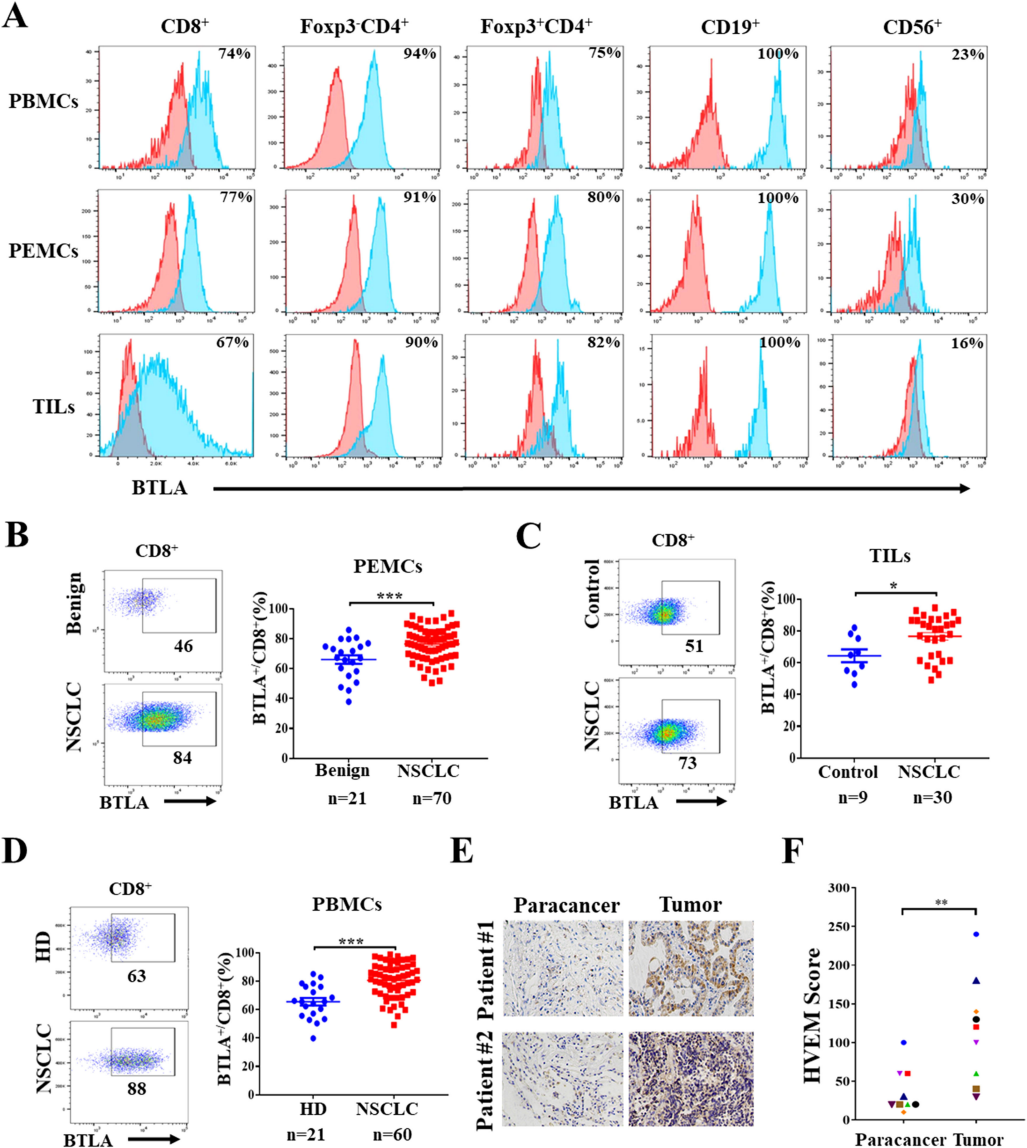
Abnormally increased infiltration of BTLA^+^CD8^+^ T cells in NSCLC tumor microenvironment **(A)** Expression of BTLA on lymphocytes in different kinds of specimens from NSCLC patient was evaluated by flow cytometry. **(B)** Representative flow plots and statistics of BTLA expression on CD8^+^ T cells from NSCLC and benign pleural effusions. **(C)** Representative flow plots and statistics of BTLA expression on CD8^+^ T cells from NSCLC and nontumor tissues. **(D)** Representative flow plots and statistics of BTLA expression on CD8^+^ T cells from NSCLC patients and health donor peripheral blood. Data are presented as mean ± SEM. *P* values were calculated by two-tailed unpaired Student’s *t*-test. **(E, F)** Expression of HVEM in tumor and paired tumor-adjacent tissues of NSCLC patients was analyzed by IHC staining, HVEM positive cells were quantified by IHC score, scale bar, 100 μm. n=9. *P* values were calculated by two-tailed paired Student’s t-test. (*, *P* < 0.05; **, *P* < 0.01; ***, *P* < 0.001).

Although previous studies have shown that BTLA acts as a negative immune checkpoint, the expression characteristics of BTLA in NSCLC microenvironment were rarely reported. In this study, we characterized BTLA expression in lymphocytes isolated from malignant pleural effusion fluid, tumor tissue and peripheral blood of NSCLC patients. Pleural effusion mononuclear cells (PEMCs), TILs and peripheral blood mononuclear cells (PBMCs) were isolated from malignant pleural effusions, tumor tissue and peripheral blood, respectively. The expression of BTLA in lymphocytes was detected by flow cytometry. In TILs, BTLA was highly expressed on approximately 67% of CD8^+^ T cells, 90% of Foxp3^-^CD4^+^ T cells and 82% of Foxp3^+^CD4^+^ T cells. In addition, BTLA was expressed on all CD19^+^ B cells and a small fraction of CD56^+^ NK cells. The distribution of BTLA expression in PEMCs and PBMCs was similar to that in PEMCs (**Figure 5A**). In addition, we analyzed the infiltration of BTLA^+^CD8^+^ T cells in specimens from NSCLC patients. We observed that the proportion of BTLA^+^CD8^+^ T cells in malignant pleural effusions (76.44 ± 1.29%, n = 70) was significantly greater than that in benign pleural effusions (64.04 ± 2.83%, n = 21) (**Figure 5B**). Similarly, the percentages of BTLA^+^CD8^+^ T cells in tumors (76.61 ± 2.41%, n = 30) and peripheral blood (80.58 ± 1.54%, n = 60) from NSCLC patients were higher than those in nontumor tissues (64.37 ± 4.09%, n = 9) and donor peripheral blood (65.53 ± 2.67, n = 21) (**Figures 5C, D**). Then, the correlation between the proportion of BTLA^+^CD8^+^ T cells and clinicopathologic features was assessed. We found that a greater proportion of BTLA^+^CD8^+^ T cells was significantly correlated with a greater CEA concentration in malignant pleural effusions (*P* = 0.014). The same relationship was found between BTLA^+^CD8^+^ T cells in tumor tissue and peripheral blood and tumor size (*P* < 0.05) (**Supplementary Tables S2-S4**). Furthermore, HVEM acts as a major ligand for BTLA, and its expression in NSCLC tumors was evaluated by immunohistochemistry. The results showed that he expression of HVEM in tumors was increased than that in paired tumor-adjacent tissues (**Figures 5E, F**) (**Supplementary Figure S1A**). In addition, HVEM was also highly expressed in NSCLC cell lines (**Supplementary Figure S1B**). Kaplan–Meier analysis revealed that NSCLC patients with high HVEM expression had significantly shorter OS and RFS than patients with low HVEM expression (**Supplementary Figure S1C**). These results suggest that BTLA^+^CD8^+^ T cells are abnormally increased in NSCLC microenvironment and that a higher proportion of BTLA^+^CD8^+^ T cells might be associated with poor prognosis.

### 3.6 CD8^+^ T cells expressing BTLA exhibit exhausted phenotype and are correlated with clinical response to anti-PD-1 therapy in NSCLC

**Figure 6:**
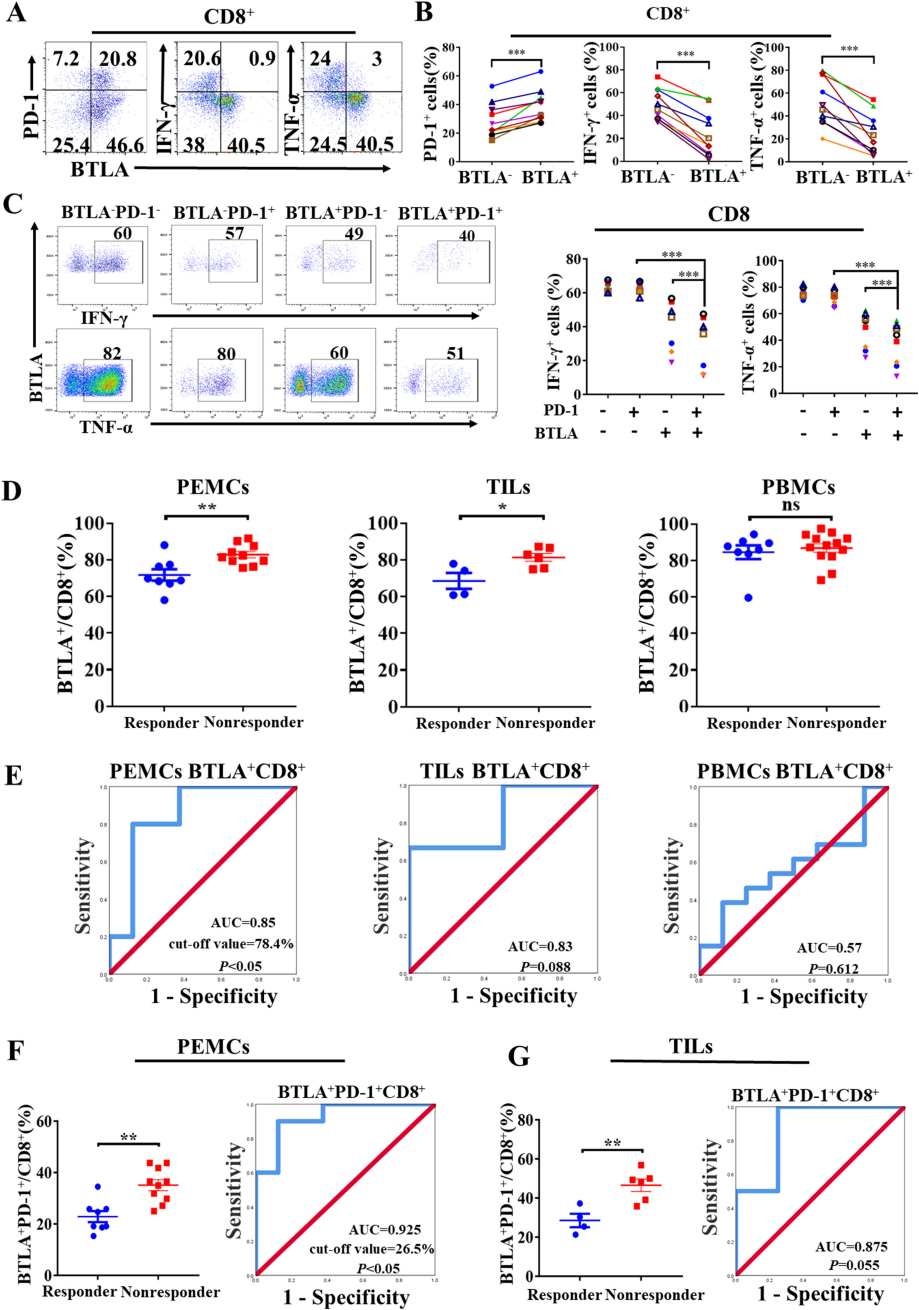
CD8^+^ T cells expressing BTLA exhibit exhausted phenotype and are correlated with clinical response to anti-PD-1 therapy in NSCLC **(A, B)** PEMCs were isolated from malignant pleural effusions. Expression of PD-1, IFN-γ and TNF-α on BTLA^-^ and BTLA^+^CD8^+^ T cells were detected by flow cytometry. n=10, paired student’s t-test was performed as statistical analysis. **(C)** Expression differences of IFN-γ and TNF-α in BTLA^-^PD-1^-^, BTLA^-^PD-1^+^, BTLA^+^PD-1^-^ and BTLA^+^PD-1^+^CD8^+^ T cells from PEMCs were analyzed. n=7. *P* values were calculated by two-tailed paired Student’s t-test. **(D)** The expression of BTLA on CD8^+^ T cells in PEMCs, TILs and PBMCs obtained from NSCLC patients sensitive or insensitive to PD-1 blockade therapy was determined by flow cytometry. *P* values were calculated by two-tailed unpaired Student’s *t*-test. **(E)** Receiver operating characteristic (ROC) analysis was performed to evaluate the predictive value of BTLA^+^CD8^+^ T cells from PEMCs, TILs and PBMCs for anti-PD-1 therapy response in NSCLC patients. **(F, G)** Frequencies of BTLA^+^PD-1^+^CD8^+^ T cells in PEMCs and TILs from NSCLC patients treated with PD-1 blockade therapy were assessed by flow cytometry. ROC analysis was performed to evaluate the predictive value of BTLA^+^PD-1^+^CD8^+^ T cells from PEMCs and TILs for anti-PD-1 treatment response in NSCLC patients. *P* values were calculated by two-tailed unpaired Student’s *t*-test. (*, *P* < 0.05; **, *P* < 0.01; ns, *P* > 0.05).

To determine the role of BTLA^+^CD8^+^ T cells in the NSCLC microenvironment, we characterized the phenotypic and functional features of BTLA^+^CD8^+^ T cells in malignant pleural effusions. We found that PD-1 was upregulated in BTLA^+^CD8^+^ T cells compared to BTLA^-^CD8^+^ T cells. BTLA^+^CD8^+^ T cells also consisted of less percentage of cells that expressed effector molecules such as IFN-γ and TNF-α than BTLA^-^CD8^+^ T cells (**Figures 6A, B**). Similar to what we observed in LLC-bearing mice, approximately 20% of CD8^+^ TILs co-expressed BTLA and PD-1 in malignant pleural effusions. Compared to BTLA^+^PD-1^-^CD8^+^ and BTLA^-^PD-1^+^CD8^+^ T cells, BTLA^+^PD-1^+^CD8^+^ T cells exhibited more impaired production of IFN-γ and TNF-α (**Figure 6C**). In addition, we investigated whether the population of BTLA^+^CD8^+^ T cells was associated with responsiveness to PD-1/PD-L1 blockade therapy in NSCLC patients. We found that the proportion of BTLA^+^CD8^+^ T cells in malignant pleural effusions and tumors from nonresponders to anti-PD-1 immunotherapy was higher than that from responders, but there was no difference in the proportion of BTLA^+^CD8^+^ T cells in peripheral blood (**Figure 6D**). Then, using receiver operating characteristic (ROC) analysis, we evaluated whether BTLA^+^CD8^+^ T cells could be a predictor of responsiveness to anti-PD-1 immunotherapy. We found that the proportion of BTLA^+^CD8^+^ T cells in malignant pleural effusions and tumors had an area under the ROC curve (AUC) greater than 0.8, and only the AUC in malignant pleural effusions was significantly different (cutoff value = 78.4%, sensitivity = 80%, specificity = 87.5%, *P* < 0.05) (**Figure 6E**). Furthermore, we evaluated the association of BTLA^+^PD-1^+^CD8^+^ T cells with response to anti-PD-1 immunotherapy in NSCLC patients. We found that the proportions of BTLA^+^PD-1^+^CD8^+^ T cells in malignant pleural effusions and tumors from nonresponders to anti-PD-1 immunotherapy were higher than those from responders. The AUC of BTLA^+^PD-1^+^CD8^+^ T cells in malignant pleural effusions and tumors was greater than that of BTLA^+^CD8^+^ T cells. However, only BTLA^+^PD-1^+^CD8^+^ T cells in malignant pleural effusions had a statistically significant AUC (cutoff value = 26.45%, sensitivity = 90%, specificity = 87.6%, *P* < 0.05) (**Figures 6F, G**). These data suggest that CD8^+^ T cells expressing BTLA in NSCLC microenvironment exhibit exhausted phenotype and are correlated with clinical response to anti-PD-1 therapy in NSCLC patients.

## 4 Discussion

The present study found that BTLA and PD-1 act synergistically to promote and maintain the CD8^+^ T cells exhausted phenotype. Combined anti-PD-1 therapy enhances the anti-tumor effect of PD-1/PD-L1 blockade by enhancing CD8^+^ T cells effector function in LLC-bearing mice. There is abnormally increased infiltration of BTLA^+^CD8^+^ T cells in NSCLC microenvironment. CD8^+^ T cells expressing BTLA in NSCLC microenvironment exhibit exhausted phenotype and are correlated with clinical response to anti-PD-1 therapy in NSCLC patients, suggesting that BTLA^+^CD8^+^ T cells might serve as biomarkers to predict the responsiveness to anti-PD-1 therapy in NSCLC patients. Our study demonstrated that BTLA blockade is a novel and promising treatment strategy for enhancing the efficacy of PD-1/PD-L1 blockade in NSCLC patients.

During tumorigenesis, various immune components, including immune checkpoints, promote the formation of an immunosuppressive tumor microenvironment to escape immune surveillance. BTLA is a lymphocyte inhibitory receptor with similarities to PD-1 and CTLA-4 (15). BTLA expression is significantly upregulated on circulating and tumor-infiltrating CD4^+^ T cells in hepatocellular carcinoma (19, 23). High expression of BTLA in T cells was correlated with advanced-stage diffuse large B-cell lymphoma (27). A greater density of tumor-infiltrating BTLA^+^CD8^+^ T cells was significantly associated with shorter overall survival and disease-free survival in gallbladder cancer patients (28). In our study, the frequency of BTLA^+^CD8^+^ T cells was significantly increased in the peripheral blood, malignant pleural effusion and tumors of NSCLC patients. BTLA^+^CD8^+^ T cells from malignant pleural effusions exhibited an exhausted phenotype with decreased cytokine production. In addition, the percentage of BTLA^+^CD8^+^ T cells in malignant pleural effusions was correlated with the CEA concentration, and the proportion of BTLA^+^CD8^+^ T cells in peripheral blood and tumors was associated with tumor size in NSCLC patients. Despite this result, it would be interesting to characterize the effect of BTLA on other lymphocytes in the NSCLC microenvironment in future studies. The expression of HVEM, the ligand for BTLA, was upregulated in NSCLC. High HVEM expression was associated with shorter OS and RFS. These findings indicate that activation of the BTLA/HVEM pathway is involved in NSCLC immune escape and results in poor prognosis.

Despite advances in immune checkpoint blockade for solid tumors, NSCLC has demonstrated resistance to anti-PD-1 therapy via multiple mechanisms of immunosuppression (29). Notably, in addition to PD-1, TILs in NSCLC exhibit upregulation of additional inhibitory immune checkpoint molecules, such as CTLA-4, TIM-3 and LAG-3 (30). As such, the use of combination checkpoint therapy involving additional checkpoint blockade agents, such as antibodies against CTLA-4, LAG-3 and TIM-3 have been explored in both preclinical and clinical investigations. In the clinical setting, the precedent for success with combination checkpoint therapy for NSCLC treatment is most salient, in which anti-CTLA-4 and anti-PD-1 combination therapy was approved by the FDA in 2016 as a first-line therapy and showed promising results of improved progression-free survival compared with anti-PD-1 therapy alone (13). In this study, BTLA expression was upregulated during treatment in LLC tumor burden mice that was not sensitive to PD-1/PD-L1 blockade. In addition, the percentage of BTLA^+^CD8^+^ T cells was increased in the malignant pleural effusions and tumors of NSCLC patient refractory to anti-PD-1 blockade therapy. BTLA^+^CD8^+^ T cells might be a potential biomarker for predicting the responsiveness of NSCLC patients to anti-PD-1 immunotherapy. Based on the above results, we hypothesize that increased proportion of tumor-infiltrating BTLA^+^CD8^+^ T cells might lead to failure of anti-PD-1/PD-L1 therapy in NSCLC and that combined anti-BTLA therapy might enhance the efficacy of anti-PD-1/PD-L1 therapy in NSCLC. Therefore, we tested anti-PD-L1 mAb in combination with anti-BTLA mAb in LLC-bearing mice. The combination of anti-PD-L1 and anti-BTLA significantly inhibited tumor growth and extended the survival of tumor-bearing mice. The mice treated with anti-PD-L1 and anti-BTLA mAbs exhibited increased infiltration of CD8^+^ and CD4^+^ T cells. CD8^+^ T cells from tumor-bearing mice administered anti-PD-L1 and anti-BTLA blockade agents exhibited increased cytotoxicity and proliferation. Similarly, previous preclinical investigations have shown that checkpoint blockade combined with PD-1 and BTLA can exert synergistic antitumor effects by directly activating CD4^+^ and CD8^+^ T cells in glioblastoma (31). Although the current study did not directly demonstrate the relationship between BTLA^+^CD8^+^ T cells and resistance to anti-PD-1/PD-L1 therapy, which requires further investigation, our results suggest that tumor-infiltrating BTLA^+^CD8^+^ T cells are potentially involved in resistance to anti-PD-1/PD-L1 blockade.

Anti-PD-1/PD-L1 therapy blocks the coinhibitory interaction between PD-1/PD-L1 in CD8^+^ TILs and subsequently induces an antitumor immune response (32, 33). Multiple immune regulatory receptors co-expressing PD-1^+^CD8^+^ TILs, such as TIM-3, LAG-3 and TIGIT have been reported (11, 34–37). TIM-3^+^PD-1^+^ TILs exhibit the most severe exhausted phenotype, as defined by failure to proliferate and produce cytokines (38). Similar to previous reports, our data indicated that BTLA^+^PD-1^+^CD8^+^ T cells in the NSCLC microenvironment exhibited a more exhausted phenotype than did BTLA^+^PD-1^-^ and BTLA^-^PD-1^+^CD8^+^ T cells. In addition, BTLA^+^PD-1^+^CD8^+^ T cells were resistant to restoring effector function by inhibiting the PD-1/PD-L1 pathway. Under combined blockade of PD-L1 and BTLA, the effector function of BTLA^+^PD-1^+^CD8^+^ T cells was significantly restored. Both PD-1 and BTLA can recruit SHP-1 and SHP-2 to inhibit the TCR signaling pathway via redundant mechanisms, which might lead to the failure of both PD-L1 and BTLA mAbs to restore the effector function of T cells (24). Anti-PD-L1 treatment did not affect the expression of PD-1 on CD8^+^ T cells but did upregulate the expression of BTLA and increase the percentage of BTLA^+^PD-1^+^CD8^+^ T cells. These findings suggested that more abundant targets are available for PD-L1 and BTLA mAbs in tumors after combination treatment. However, the mechanism by which anti-PD-L1 treatment regulates BTLA expression needs to be further determined. This may be related to the increased release of cytokines such as IL-10 and IL-6 in tumor microenvironment that can promote BTLA expression after PD-1/PD-L1 blockade (39).

In this study, the limited sample size for clinical observation potentially caused selection bias. Second, because the main clinical first-line treatment for NSCLC is anti-PD-1 therapy combined with chemotherapy, we could not analyze the correlation between the responsiveness to anti-PD-1 or anti-PD-L1 monotherapy and the infiltration of BTLA^+^CD8^+^ T cells into tumors. Furthermore, the molecular mechanisms by which BTLA and PD-1 synergistically maintain the exhausted phenotype of CD8^+^ T cells and by which combination therapy reverses BTLA^+^PD-1^+^CD8^+^ T cells have not been investigated. However, the current clinical observations and preclinical findings suggest that dual BTLA and PD-L1 blockade is a promising combination therapy strategy for NSCLC.

## 5 Conclusion

Collectively, our study showed that BTLA and PD-1 cooperatively inhibit the activity of CD8^+^ T cells and are associated with resistance to PD-1/PD-L1 pathway blockade in NSCLC patients and that anti-BTLA blockade enhances the antitumor efficacy of anti-PD-L1 blockade by reversing the exhausted phenotype of BTLA^+^PD-1^+^CD8^+^ T cells, which suggests that dual BTLA and PD-1/PD-L1 blockade should be further explored to elicit potent antitumor CD8^+^ T-cell responses in NSCLC patients.

## Data Availability

The original contributions presented in the study are included in the article/**Supplementary Material**. Further inquiries can be directed to the corresponding author/s.
