# Supplementary material for: B and T lymphocyte attenuator (BTLA) and PD-1 pathway dual blockade promotes antitumor immune responses by reversing CD8+ T-cell exhaustion in non-small cell lung cancer
Source: Front Immunol. 2025 May 20;16:1553042. doi: 10.3389/fimmu.2025.1553042 (PMC12129974; doi:10.3389/fimmu.2025.1553042)
Supplement: Supplementary file 1 [file DataSheet1.pdf]

Supplemental Table S1 Characteristics of patients with NSCLC whose specimens were collected

Variables	Peripheral blood (n, %)	Pleural effusion (n, %)	Tumor (n, %)
Age（years）			
<60	19 (32)	17 (24)	9 (30)
≥60	41 (68)	53 (76)	21 (70)
Gender			
Male	47 (78)	48 (69)	24 (80)
Female	13 (22)	22 (31)	6 (20)
Histology			
Squamous	20 (33)	8 (11)	9 (30)
Non-Squamous	40 (67)	62 (89)	21 (70)
Tumor diameter			
≤3 cm	6 (10)	4 (6)	3 (10)
＞3 cm	54 (90)	66 (94)	27 (90)
Lymph node metastasis			
No	12 (20)	5 (7)	8 (27)
Yes	48 (80)	65 (93)	22 (73)
Stage			
I-II	9 (15)	0 (0)	5 (17)
III-IV	51 (85)	70 (100)	25 (83)
Anti-PD-1 antibody			
Untreated	39 (65)	52 (74)	20 (67)
Responder	8 (13)	8 (12)	4 (13)
Nonresponder	13 (22)	10 (14)	6 (20)
