# Supplementary material for: B and T lymphocyte attenuator (BTLA) and PD-1 pathway dual blockade promotes antitumor immune responses by reversing CD8+ T-cell exhaustion in non-small cell lung cancer
Source: Front Immunol. 2025 May 20;16:1553042. doi: 10.3389/fimmu.2025.1553042 (PMC12129974; doi:10.3389/fimmu.2025.1553042)
Supplement: Supplementary file 2 [file DataSheet2.pdf]

Supplemental Table S2 Correlation between the proportion of $BTLA^{+}CD8^{+}$ T cells and clinicopathologic features in malignant pleural effusion of NSCLC patients

Characteristic	n (%)	$BTLA^{+}/CD8^{+}$（%）	*P* value
Age（years）			
<60	17 (24)	76.43±9.07	0.753
≥60	53 (76)	74.32±2.38	
Gender			
Male	48 (69)	77.16±2.84	0.128
Female	22 (31)	69.34±3.95	
Histology			
Squamous	8 (11)	71.5±2.63	0.693
Non-Squamous	62 (89)	75±2.63	
Tumor diameter (cm)			
≤3	4 (6)	73.73±8.6	0.889
＞3	66 (94)	74.78±2.53	
Lymph node metastasis			
No	5 (7)	73.23±6.24	0.798
Yes	65 (93)	74.89±2.51	
CEA (ng/ml)			
≤20	15 (21)	70.84±3.92	0.014
>20	55 (79)	81.9±1.98	
