# Supplementary material for: B and T lymphocyte attenuator (BTLA) and PD-1 pathway dual blockade promotes antitumor immune responses by reversing CD8+ T-cell exhaustion in non-small cell lung cancer
Source: Front Immunol. 2025 May 20;16:1553042. doi: 10.3389/fimmu.2025.1553042 (PMC12129974; doi:10.3389/fimmu.2025.1553042)
Supplement: Supplementary file 3 [file DataSheet3.pdf]

Supplemental Table S3 Correlation between the proportion of BTLA$^+$CD8$^+$ T cells in TILs and clinicopathologic features

Characteristic	n (%)	BTLA$^+$/CD8$^+$（%）	*P* value
Age（years）			
<60	9 (30)	84.59±3.69	0.2412
≥60	21 (70)	83.29±2.93	
Gender			
Male	24 (80)	87.22±3.04	0.336
Female	6 (20)	82.48±3.72	
Histology			
Squamous	9 (30)	86.16±6.45	0.434
Non-Squamous	21 (70)	87.06±2.97	
Tumor diameter (cm)			
≤3	3 (10)	71.48±3.84	0.024
＞3	27 (90)	82.43±2.28	
Lymph node metastasis			
No	8 (27)	84.4±5.12	0.423
Yes	22 (73)	80.23±3.76	
Stage			
I-II	5 (17)	86.4±2.38	0.273
III-IV	25 (83)	80.31±2.85	
