# Supplementary material for: B and T lymphocyte attenuator (BTLA) and PD-1 pathway dual blockade promotes antitumor immune responses by reversing CD8+ T-cell exhaustion in non-small cell lung cancer
Source: Front Immunol. 2025 May 20;16:1553042. doi: 10.3389/fimmu.2025.1553042 (PMC12129974; doi:10.3389/fimmu.2025.1553042)
Supplement: Supplementary file 4 [file DataSheet4.pdf]

Supplemental Table S4 Correlation between the proportion of $BTLA^{+}CD8^{+}$ T cells and clinicopathologic features in peripheral blood of NSCLC patients

Characteristic	n (%)	$BTLA^{+}/CD8^{+}$（%）	*P* value
Age（years）			
<60	19 (32)	86.82±3.59	0.256
≥60	41 (68)	79.75±3.24	
Gender			
Male	47 (78)	80.66±3.08	0.531
Female	13 (22)	84.95±5.09	
Histology			
Squamous	20 (33)	88.96±2.17	0.812
Non-Squamous	40 (67)	86.7±3.53	
Tumor diameter (cm)			
≤3	6 (10)	74.98±3.92	0.015
＞3	54 (90)	86.44±2.19	
Lymph node metastasis			
No	12 (20)	89.4±5.21	0.218
Yes	48 (80)	80.12±2.88	
Stage			
I-II	9 (15)	87.3±4.65	0.438
III-IV	51 (85)	81.91±2.78	
