# Supplementary material for: B and T lymphocyte attenuator (BTLA) and PD-1 pathway dual blockade promotes antitumor immune responses by reversing CD8+ T-cell exhaustion in non-small cell lung cancer
Source: Front Immunol. 2025 May 20;16:1553042. doi: 10.3389/fimmu.2025.1553042 (PMC12129974; doi:10.3389/fimmu.2025.1553042)
Supplement: Supplementary file 5 [file DataSheet5.pdf]

Supplemental Figure S1

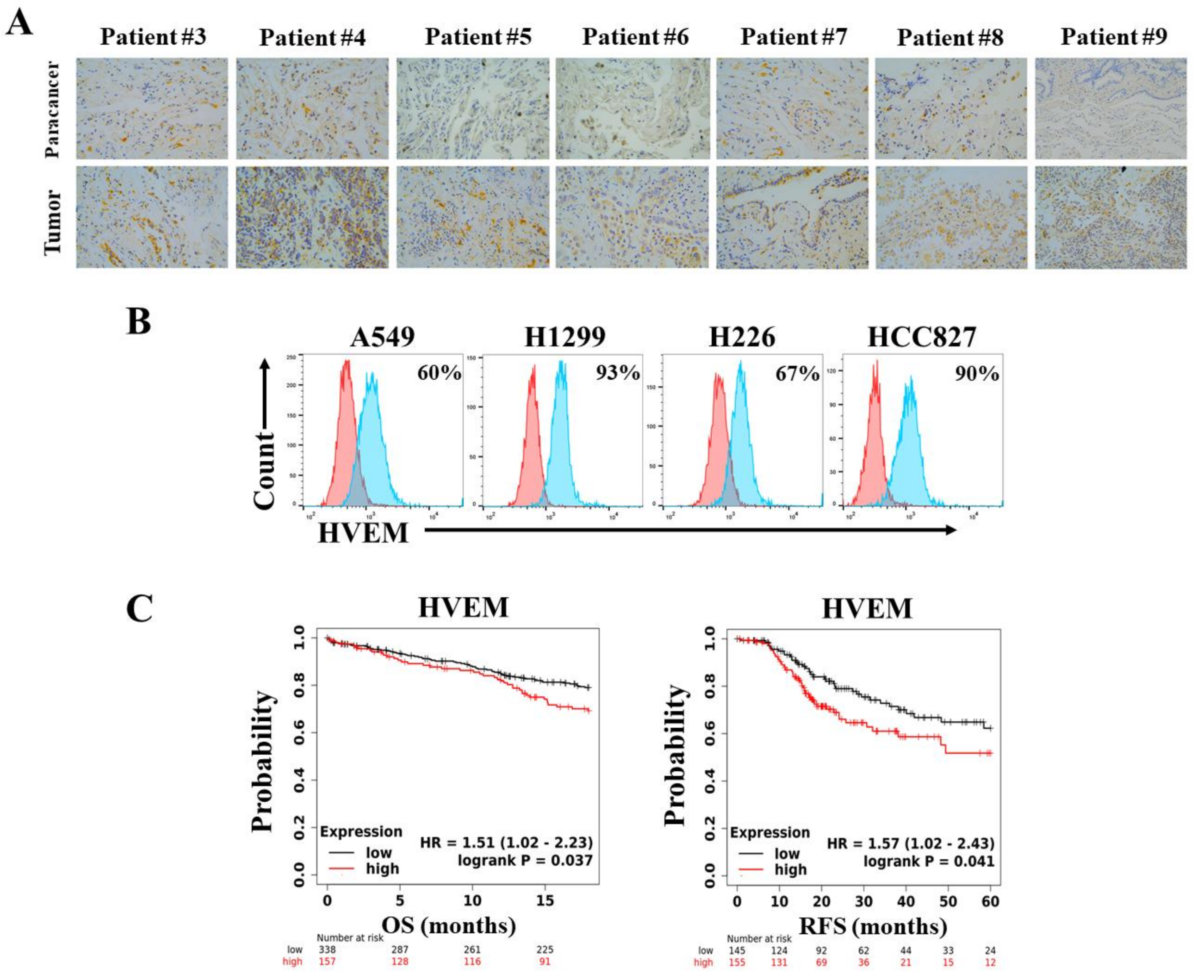

High expression of HVEM in NSCLC was associated with poor prognosis. (A) Expression of HVEM in tumor and paired tumor-adjacent tissues of NSCLC patients was analyzed by IHC staining. **(**B) NSCLC cell lines (A549, H1299, H226 and HCC827) expressed HVEM. (C) Kaplan-Meier survival curves stratified by HVEM expression from NSCLC patients in TCGA database.
